# Anticancer Activities of Citrus Peel Polymethoxyflavones Related to Angiogenesis and Others

**DOI:** 10.1155/2014/453972

**Published:** 2014-08-28

**Authors:** Liwen Wang, Jinhan Wang, Lianying Fang, Zuliang Zheng, Dexian Zhi, Suying Wang, Shiming Li, Chi-Tang Ho, Hui Zhao

**Affiliations:** ^1^Tianjin Key Laboratory of Food and Biotechnology, School of Biotechnology and Food Science, Tianjin University of Commerce, Tianjin 300134, China; ^2^Institute of Radiation Medicine, Chinese Academy of Medical Sciences and Peking Union Medical College, Tianjin 300192, China; ^3^Hubei Key Laboratory of Economic Forest Germplasm Improvement and Resources Comprehensive Utilization, Huanggang Normal University, Huanggang, Hubei 438000, China; ^4^Department of Food Science, Rutgers University, New Brunswick, NJ 08901-8502, USA; ^5^Department of Hematology and Translation Medicine Centre, Hebei Union University Affiliated Hospital, Tangshan, Hebei 063000, China

## Abstract

Citrus is a kind of common fruit and contains multiple beneficial nutrients for human beings. Flavonoids, as a class of plant secondary metabolites, exist in citrus fruits abundantly. Due to their broad range of pharmacological properties, citrus flavonoids have gained increased attention. Accumulative in vitro and in vivo studies indicate protective effects of polymethoxyflavones (PMFs) against the occurrence of cancer. PMFs inhibit carcinogenesis by mechanisms like blocking the metastasis cascade, inhibition of cancer cell mobility in circulatory systems, proapoptosis, and antiangiogenesis. This review systematically summarized anticarcinogenic effect of citrus flavonoids in cancer therapy, together with the underlying important molecular mechanisms, in purpose of further exploring more effective use of citrus peel flavonoids.

## 1. Introduction

In our daily diet, the average intake of flavonoids of every day ranges from 150 mg to 300 mg [1]. As the primary source, flavonoids from citrus fruit or juice take up to 10%, of which juices and fruits offer 8 mg and 3 mg, respectively [2]. The main components in citrus possess phenols, amino acids, essential oils, pectin, carotenoids, flavonoids, and vitamin C. Although flavonoids are generally considered to be nonnutritive agents, interest in flavonoids has arisen because of their potential role in the prevention of major chronic diseases. Flavonoids are polyphenolic compounds and include a phenyl benzopyrone structure, representing as two benzene rings (C6) joined by a linear three-carbon chain (C3), with a carbonyl group at the C_4_ position. The citrus flavonoids include a class of glycosides, namely, hesperidin and naringin, and another class of O-methylated aglycones of flavones such as nobiletin and tangeretin, which are relatively common two polymethoxylated flavones (PMFs) [3]. PMFs exist almost ubiquitously in citrus plants. Six PMFs and three major 5-demethoxyflavones can be extracted from a variety of citrus peels. The wide biochemical functions of flavonoids in orange peel have been studied extensively recently. They increased serum antioxidant capacity against lipid peroxidation [4] and reduced the elderly oxidative stress. These compounds also performed beneficial effects of anti-inflammation, antitumor [5, 6], and antiatherosclerosis [7]. Meanwhile, they serve as supplementary of drug chemotherapy [8], diabetes health food [9], and neuroprotective drug [10].

In recent years, epidemiological studies have shown that there is a connection that flavonoid intake may reduce the risk of developing colon cancer [11, 12]. Moreover, it may prevent men against Parkinson's disease (PD) after identifying 805 participants (438 men and 367 women) who developed PD during 20–22 years of follow-up [13], help women get out of the risk of gastric cancer and breast cancer with 10% reduction in risk of breast cancer associated with high intake of citrus fruits [14], and reduce the possibility of ischemic stroke during 14 years of follow-up, confirmed with 1803 incident strokes. After adjusting for potential confounders, women in the highest compared with the lowest quintile of flavanone intake had a relative risk of ischemic stroke of 0.81 [15].

Cancer is the life threatening and dreadful disease characterized by the abnormal proliferation of cells that invade the adjacent tissues and cause the destruction of these tissues. It is the second leading cause of death all over the world. More than six million deaths each year occurring in the world are due to cancer. Several lines of evidence indicated that tumorigenesis in humans is a multistep process and these steps reflect genetic alterations that drive the progressive transformation of normal human cells into highly malignant derivatives [16]. Conventional treatment chemotherapy could cause adverse and toxic side effects on normal cells while curing cancer and therefore fails to control the disease. The alternative solution for the harmful effects of synthetic agents is the use of natural plants, which provide outstanding contribution to modern therapeutics [17]. It has been shown by clinical studies and phytochemical investigation currently that many herbs exhibit antitumor potential. In this review, we center on the latest research progress on the antitumor activities of citrus peel compounds.

## 2. Anticarcinogenic Properties

**Figure 1:**
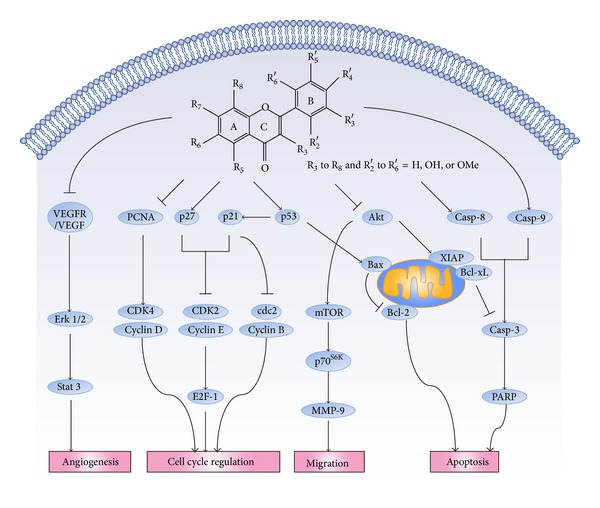
Polymethoxyflavones exert beneficial effects through antigrowth, antiangiogenesis and cell cycle arrest commands or mediate signals to live or die by apoptosis. At one level, this depiction is simplistic, as different cancer cells are exposed to a specific complex microenvironment, each of these pathways regulated by PMFs is connected with signals originating from other cells in the tumor microenvironment. Schematic representation of the main molecular mechanisms of flavonoids in citrus peel on anticancer.

In the tumor microenvironment, from cancer cells initiation to promotion and eventually progression, compelling evidence indicates the potential activities of flavonoids in citrus peel cover inhibiting oncogenesis, proliferation, neovascularization, and metastasis and inducing apoptosis.  Figure 1 schematizes the main anticarcinogenic pathways of citrus peels flavonoids and different bioactivity aspects of specific compounds stated in this review are summarized in Table 1.

### 2.1. Cell Cycle Arrest

**Figure 2:**
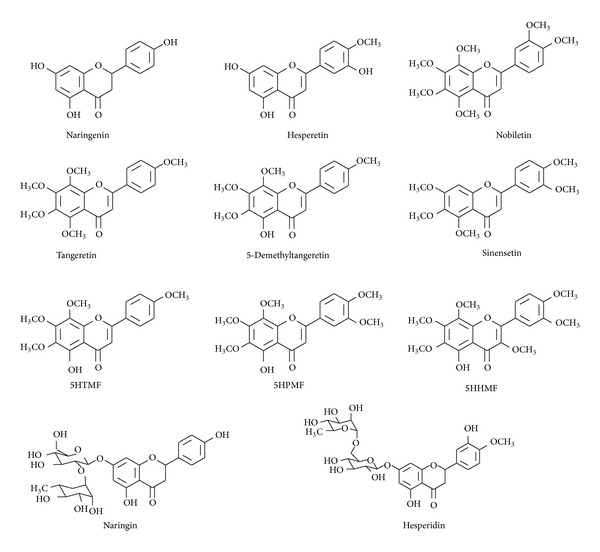
The chemical structures of citrus peel flavonoids molecules that are discussed in this paper.

Cell cycle abnormalities are closely associated with cancer, and citrus peel flavonoids substantially influence on cell cycle arrest. Cell cycle is an important regulatory mechanism of cell growth, development, and differentiation. In mammals, the cell cycle comprises the G1, S, G2, and M phases. Cell cycle checkpoints keep the maintenance of genomic integrity by inhibiting damaged or incomplete DNA. G2/M checkpoint ensures that the cells do not initiate mitosis before repairing damaged DNA after replication. The cell cycle progression depends on a cascade of enzymes by sequential activation and inactivation of cyclin, cyclin-dependent kinases (CDKs), and cyclin-dependent kinase inhibitors (CDKIs) [18]. Cdc2 interacts with cyclin B1 and forms a cdc2-cyclin complex. The G2/M transition is regulated by the sequential activation and inactivation of the cdc2/cyclin B complex [19]. Non-small-cell lung cancer (NSCLC) A549 cells arrest and apoptosis can be induced by flavonoids extracted from Korean* Citrus aurantium* L. [20]. Downregulation of cdc2, cdc25c, and cyclin B1 and the upregulation of p21 resulted in G2/M arrest in A549 cell line. Actually, the mechanisms of parts of flavonoids components are being further explained. As one of the most prevalent flavonoids extracted from orange, hesperetin repressed CDK2, CDK4, and cyclin D and simultaneously enhanced p21 and p27 expression to block cell cycle in G1 phase [21]. It is also reported that hesperetin and naringenin exhibited the same results in cervical cancer cell SiHa and liver cancer cell HepG2, respectively [22, 23]. In human breast and colon cancer cells, both tangeretin and nobiletin inhibited the proliferation and led to accumulation of cells in the G1/S cell cycle compartment and did not involve induction of cell death or apoptosis. This finding may provide advantageous theory basis in treating tumors as it would restrict proliferation in a manner less likely to induce cytotoxicity and death in normal tissues [24]. More recently, the study on derivatives of citrus peel flavonoids causes widely concern as well. For instance, in all three NSCLC cells A549, H460, and H1299, 5-demethyltangeretin mediated G2/M cell cycle arrest by upregulating p53 and p21^Cip1/Waf1^ and downregulating cdc2 and cyclin B1 [25]. Among three 5-hydroxy polymethoxyflavones (5OH-PMFs), 5-hydroxy-3,6,7,8,3′,4′-hexamethoxyflavone (5HHMF), 5-hydroxy-6,7,8,3′,4′-pentamethoxyflavone (5HPMF), and 5-hydroxy-6,7,8,3′,4′-pentamethoxyflavone (5HTMF), the data showed that the 5HTMF-induced G0/G1 arrest was the most responsive to the change of the p21 and p53 status of the colon cancer cells, indicating the essential role of the 4′-methoxyl group on B ring of 5HTMF in inducing cell cycle arrest [26]. The chemical structures of flavonoids in citrus peel can be found in Figure 2. Results above inevitably support the idea that specific structural elements of the flavonoids are the key determinants of pharmacological activities.

### 2.2. Suppression of Proliferation and Proapoptosis

One of the most basic features of cancer cells is their ability to proliferate chronically. Apart from blocking cell cycle, flavonoids in citrus peel can also inhibit cell proliferation and promote apoptosis, especially in triple-negative (ER-/PR-/HER2-) breast cancer (TNBC). PMFs triggered influx of Ca^2+^ and mobilization of intracellular Ca^2+^ store, accompanied by activation of calpain and caspase-12 [27]. There are further researches on mechanisms of these functions. Crude methanol extracts of the peels of* Citrus aurantium* L. induced caspase-dependent apoptosis through Akt pathway by inhibiting expression of XIAP and Bcl-2 which are antiapoptotic proteins, providing the fact that they have anticarcinogenic activity on human leukemia cells U937 [28]. In another leukemia cell line NALM-6, hesperidin, as the glycoside of hesperetin, promoted apoptosis via conducting the expression of p53 and peroxisome proliferator-activated receptor gamma (PPAR *γ*) and suppressing the activation of NF-*κ*B [29]. Tangeretin-induced caspase-3 activation and elevated surface phosphatidylserine exposure demonstrated tangeretin apoptosis-inducing activity in LoVo/Dx cells, which might also enhance multidrug-resistance [30]. In HCT116 human colon cancer cells, 5-hydroxy polymethoxyflavones (5OH-PMFs), especially 5HHMF and 5HTMF, induce cellular apoptosis in human colon cancer cells by p53- and Bax-dependent mechanisms [26]. Noteworthy, by looking for relationships between chemical profiles and cell viability profiles, cytotoxic effects as indicated by a decrease of IC_50_ values with increasing concentration of OH-PMFs were observed in different orange peel extracts [31]. Subsequent data showed that when MCF-7 breast cancer cells were treated with PMF and hydroxylated PMF separately, effective concentrations of hydroxylated PMFs in inhibiting growth, inducing apoptosis, and increasing intracellular Ca^2+^ were lower than those of nonhydroxylated PMFs [32]. These already available results may offer a conclusion that OH-PMFs have better potential cytotoxic effect.

In the research of mice, oral feeding of gold lotion (GL), a formulated product made from the peels of six citrus fruits, decreased the number of aberrant crypt foci (ACF) in mice colonic tissues [33]. This compound is rich in flavonoids with a total measured content of at least 450 ppm or 0.45 mg/mL; its PMFs content is as high as 106 ppm or 0.1 mg/mL. Due to its high content of flavonoids, it has also been proven to inhibit the nuclear translocation of NF-*κ*B into the nucleus [34]. Similarly, hesperetin has potential effect on proliferation of cancer cell in vivo. For 1,2-dimethylhydrazine- (DMH-) induced colon cancer model in rats, it exerted significant inhibitory effect on proliferating cell nuclear antigen in ACF [35]. Moreover, hesperetin inhibited growth of aromatase-expressing MCF-7 tumor in ovariectomized athymic mice by reducing cyclin D1, CDK4, and Bcl-x(L), while upregulating the level of p57^Kip2^ [36]. Data above provided supporting evidence that flavonoids from citrus peel could suppress carcinogenesis in vivo.

Our latest research showed that, in MCF-7 human breast cancer cells, 5-acetyl-6,7,8,4′-tetramethylnortangeretin (5-ATAN), which replaces the methyl groups of tangeretin with acetyl groups at the C5 position of tangeretin, showed more powerful abilities than its parent compound. Then, we looked for evidence of 5-ATAN on apoptosis. Translocation of apoptosis-inducing factor (AIF) and phosphorylation of H2AX are commonly used for evaluating the impact of natural compounds-induced caspase-independent apoptosis pathway [37, 38]. Our results clearly supported the notion that proapoptosis of 5-ATAN acted through caspase-independent mechanisms in case AIF translocation and H2AX phosphorylation took place in MCF-7 cells when 5-ATAN was added. Allowing for apoptotic extrinsic pathway, no clear evidence had been found about activation of caspase-8, cleavage of BID, and regulation of FADD, indicating extrinsic pathway was not required under this circumstance. Strikingly, we also found that increase of Bax/Bcl-2 ratio, Δ*ψ*m dissipation, release of cytochrome C, and cleavage of caspase-9 after exposure to 5-ATAN in a time-dependent manner which indicated caspase-dependent intrinsic pathway were also required in the MCF-7 cells [39]. All these researches together point to a possible protective effect of citrus flavonoids and their derivatives against sustained proliferation of cancer cells.

### 2.3. Combined Chemotherapy

Traditional treatment of cancer has been facing a huge number of problems, in view of its complex molecular pathophysiology that varies according to each type. Several ways in the treatment of breast cancer have been developed that are surgery, chemotherapy, hormonal therapy, and radiation. Doxorubicin, a chemotherapeutic agent commonly used in breast cancer treatment, showed low effectivity, rendering its resistance and toxicity on normal tissues [40]. An approach in overcoming such problem is the development of agents used in combination with chemotherapeutic agents to lead to better result. Cochemotherapy may increase chemotherapeutic agents' efficacy, allowing the use of lower dosage of chemotherapeutic agent, resulting in the decrease of toxicity on normal tissues compared to chemotherapeutic agent alone [41]. In terms of medicine, hesperidin, tangeretin, and nobiletin could all improve doxorubicin cytotoxic chemotherapy [8]. When combining concentration of 200 nM doxorubicin and 100 *μ*M hesperidin together in treating with MCF-7 cells, they increased cytotoxic effect, modulated cell cycle, and induced apoptosis of MCF-7 cells [42]. Meanwhile, tangeretin synergistically increased the cytotoxic effect of doxorubicin by inducing cells death and arresting cell cycle's phase both in MCF-7 and T47D breast cancer cells. Different from tangeretin, nobiletin increased doxorubicin's cytotoxic activity in MCF-7 cells, but not in T47D cells [8]. Cyclophosphamide is a cytotoxic alkylating drug with a high therapeutic index and is effective against a variety of cancers. Despite its effectiveness for the treatment of cancer, it induces a wide range of adverse side effects and toxicity, such as nausea, vomiting, and hematopoietic toxicity, which limit the use of this drug in clinic. In animal experiments, hesperetin can decrease the genotoxic effect of mice bone marrow cells when synergistically functioned with cyclophosphamide [43].

### 2.4. Anticancer Metastasis

Invasion and metastasis are a multistep process and are described as a series of discrete steps, usually called invasion—the metastatic cascade [44]. It describes a process of continuous change of cell biology, local invasion from the beginning, followed by intravasation into surrounding blood and lymphatic vessels, and transit and extravasation of cancer cells through the lymph or blood transport system and lumina vessels, then cancer nodules formation, and finally into the solid tumor growth. Metastasis of malignant tumors and proliferation of vascular smooth muscle (VSMC) are greatly related to inflammatory cell adhesion. As for matrix metallopeptidase-2 (MMP-2) and metallopeptidase-9 (MMP-9), they contribute greatly to tumor metastasis and invasion and are considered to be predictive markers for cancer. Naringin, a major flavonoid extracted from grapefruit and other citrus fruits, suppressed the upregulation of MMP-9 and repressed the PI3K/AKT/mTOR signaling pathway. Furthermore, naringin suppressed TNF-a-mediated release of interleukin-6 and interleukin-8 (IL-6 and IL-8) [45]. AKT, a serine/threonine protein kinase, is a downstream target of PI3K and it plays a pivotal role in cell migration, growth, and antiapoptotic events in various types of cells [46]. Tangeretin inhibited platelet-derived growth factor- (PGDF-) BB-induced proliferation and migration of aortic smooth muscle cells by blocking AKT activation in a dose-dependent manner [47]. As the aglycone moiety of naringin chemical structure, naringenin, induced heme oxygenase-1 (HO-1) expression and subsequently decreased ROS generation and VSMC activation induced by TNF-*α* [48]. Besides, in human prostate tumor xenograft mouse model, intraperitoneal injection or oral administration GL can downregulate MMP-2 and MMP-9 protein expression levels and dramatically reduce both the weights (57%–100% inhibition) and volumes (78%–94% inhibition) of the tumors without any observed toxicity in the meantime [49].

### 2.5. Antiangiogenesis

Angiogenesis is a physiological process of forming new blood vessels from preexisting vessels, which involves the induction of new sprouts, coordinated and directed endothelial cell migration, proliferation, sprout fusion, and lumen formation [50]. Similar to normal tissue, tumors need supplies like nutrients and oxygen. They also need to remove metabolic waste. Tumor-associated neovasculature delivers these needs. In fact, angiogenesis is essentially required at almost every step of tumor progression and metastasis. In some physiological processes such as wound repair, angiogenesis starts only in the adult temporarily. Oppositely, in tumor growth, angiogenic switch is almost always activated and continuing to generate new blood vessels, which in turn support the tumor growth [51]. Tumor angiogenesis is a complex process and involves the crosstalk of tumor cells, endothelial cells, phagocytes, and their secreted factors, which may act as promoters or inhibitors of angiogenesis [52]. So, a balance between proangiogenic and antiangiogenic growth factors and cytokines tightly controls angiogenesis.

As one of the angiogenesis inducers, vascular endothelial growth factor-A (VEGF) can be used as a marker of angiogenesis.* VEGF-A* gene encodes the ligand involved in neovascularization during the embryonic and neonatal development, homeostasis, and survival of endothelial cells, as well as physiological and pathological state of adult [50]. When mice with human prostate tumor xenograft were intraperitoneally injected with GL, the protein expression level of VEGF was suppressed significantly [49]. In addition, oral administration of GL strongly and dose dependently reduced the protein levels of VEGF in AOM-induced colonic tissues. GL suppressing the ACF formation might be through inhibiting the colonic mucosa cellular proliferation and angiogenesis [33].

Through combined inhibition of multiple angiogenesis-related endothelial cells (EC) functions, nobiletin had been demonstrated to have concentration-dependent inhibitory effects on angiogenic differentiation induced by VEGF and FGF (fibroblast growth factors). In a chick embryo chorioallantoic membrane assay, nobiletin showed an antiangiogenic activity with the ID_50_ value being 10 lg (24.9 nmol) per egg [53].

With human umbilical vein endothelial cells (HUVECs) in vitro and zebrafish in vivo models, PMFs showed different degrees of potency of antiangiogenesis activity. Sinensetin, which showed the most potent antiangiogenesis activity and the lowest toxicity, inhibited angiogenesis by inducing cell cycle arrest in the G0/G1 phase in HUVEC culture and downregulating the mRNA expressions of angiogenesis genes* flt1*,* kdrl*, and* hras* in zebrafish [54]. Nobiletin differs from sinensetin by having methylation at the C8 position. Together with previous research of nobiletin [55], structure-activity relationship analysis indicated that the absence of a methoxylated group at the C8 position offers lower lethal toxicity in addition to enhancing the antiangiogenesis activity via observing intersegmental vessel development in zebrafish embryos [54].

### 2.6. Scavenging of ROS

Flavonoids also exert their chemopreventative effect via inhibition of certain phase I metabolizing enzymes, such as cytochrome P450 which metabolically activates a large number of procarcinogens triggering carcinogenesis. The chemopreventative effects of flavonoids are closely linked to their anticancer properties that involve the scavenging of reactive oxygen species (ROS) and growth promoting oxidants which are the major catalysts for tumor promotion. Tangeretin, a polymethoxylated flavone, can inhibit cancer cell proliferation by improving antioxidant properties such as decreasing the levels of lipid peroxide, enzymatic antioxidants SOD, CAT, and GPx, and nonenzymatic antioxidants such as GSH, vitamin C, and vitamin E in 7,12-dimethyl benz(a)anthracene (DMBA) induced mammary carcinoma in rats [56]. Otherwise, the propensity of a flavonoid to inhibit free radical mediated events is governed by its chemical structure. Specific structural elements of the flavonoids determinate antioxidation activity of these compounds. Free radical scavenging capacity is primarily attributed to the high reactivity of hydroxyl substituents. Flavonols and flavanols with a 3-OH group both have planarity, which increased flavonoid phenoxyl radical stability correspondingly [57]. Furthermore, methoxy groups introduce unfavorable steric effects and increase lipophilicity and membrane partitioning. A double bond and carbonyl function in the heterocycle or polymerization of the nuclear structure increased activity by affording a more stable flavonoid radical through conjugation and electron delocalization [58]. Remarkably, glycosylation of flavonoids reduced their in vitro antioxidantive activity compared to the corresponding aglycones [59]. The same results were observed in O-methylated flavonoids, which showed weaker antioxidation than their respective aglycones [60]. As an example, 5,3-didemethylnobiletin showed much stronger inhibitory effect on human colon cancer cell growth than 5-demethylnobiletin by cell viability assay [61]. These correlations between the flavonoid structure and their free radical scavenging activity need to be further investigated for better understanding and clinical application.

## 3. Pharmacokinetics of PMFs and Cancer Therapy

Pharmacokinetics describes how the body affects a specific drug after administration through mechanisms of absorption and distribution, as well as the chemical changes of the substance in the body. At a practical level, a drug's bioavailability can be defined as the proportion of the drug that reaches its site of action. Poor absorption and extensive conjugative metabolisms greatly limit bioavailability of dietary flavonoids.

### 3.1. PMF's Bioavailability

The bioavailability is an overall effect of absorption, distribution, metabolism, and excretion and plays an important role in dictating cancer preventive efficacy of dietary components in humans. Bioavailability testing can be divided into in vitro and in vivo bioavailability. In vitro bioavailability test can be a good predictor of the latter one.

Currently, human colon adenocarcinoma cell line caco-2 cell model is established to simulate the human intestinal absorption so as to test permeability and study absorption mechanism. The caco-2 data of 3′-hydroxy-5,6,7,4′-tetramethoxyflavone, 3,5,6,7,8,3′,4′-heptamethoxyflavone, and 3-hydroxy-5,6,7,8,3′,4′-hexamethoxyflavone showed superb permeability [62]. Meanwhile, the lyophilisation solubility assay (LYSA), a rapid method to test drugs and active nutrients compounds, was adapted to measure the solubility of PMFs. The solubility data showed that hydroxylated PMFs were better than their fully methoxylated counterparts. Considering the solubility and permeability together, the overall high absorption of PMFs contributes to their good bioavailability.

Also, in NSCLC A549 cell line, 5-hydroxylated PMFs had much stronger inhibitory effects on cancer cells in comparison with their permethoxylated counterparts, for IC_50_ value of 5-demethyltangeretin (5DT) was 78.9-fold lower than that of tangeretin. Since cancer cells can pump the cytotoxic agents out via overexpression of multidrug resistant efflux proteins, cells were treated with 5DT or tangeretin at the same concentration. HPLC analysis revealed that the intracellular levels of 5DT in NSCLC cells were 2.7–4.9-fold higher than those of TAN. This suggested that NSCLC cells may have better uptake efflux of 5DT compared with TAN. Additionally, molecular structure showed that 5DT had higher lipophilicity than tangeretin [63]. High lipophilicity could enhance 5DT binding to the plasma membrane, which in turn could promote the uptake of 5DT into cytosol of the cancer cells.

### 3.2. PMFs and Metabolites in Cancer Therapy

Biotransformation of dietary components is crucial for their in vivo biological activities after oral ingestion because the process of drug metabolism notably influences drugs effects and toxicity. In the research of nobiletin metabolites, by comparing supercritical fluid chromatography (SFC) profiles of metabolite mixtures with the synthesized standard compounds, three major metabolites were proved to be 4′-demethylnobiletin, 3′-demethylnobiletin, and 3′,4′-didemethylnobilietin in mouse urine [64]. Further research had demonstrated that 3′,4′-didemethylnobilietin exhibited greater bioactivities than nobiletin. As another example, it has been confirmed that 5-demethylnobiletin had strong antiproliferative effects on cancer cells. Thus, urine samples were collected from mice fed with 5-demethylnobiletin and processed for HPLC-ESI-MS analysis. Three major metabolites were characterized as 5,3′-didemethylnobiletin, 5,4′-didemethylnobiletin, and 5,3′,4′-tridemethylnobiletin. Cell viability assay in human colon cancer cells demonstrated that these three metabolites showed IC_50_ of 0.12, 5.5, and 4.2 *μ*M in SW620 cells, while 5-demethylnobiletin at 10 *μ*M only caused 37% inhibition [61]. Hence, it can be concluded that PMFs in citrus peels may produce much stronger active anticancer compounds through biotransformation.

## 4. Conclusions

Taken all together, a considerable number of well-established lines of evidence have confirmed that flavonoids in citrus peel exhibit a remarkable spectrum of efficacious biological activities, particularly in antitumorigenesis. Excellent permeability through membrane allows citrus flavonoids to possess great bioavailability which consequently attracts researchers to perform scientific studies for effective disease prevention and treatment. There are more modified flavonoids in citrus peel being investigated, which could offer help to improve dose-effect relationship greatly and advance the security and stability of compounds.

## Figures and Tables

**Table 1:** Anticarcinogenic activity of citrus polymethoxyflavonoids and their derivatives.

Polymethoxyflavone	Functions	Mechanisms	References
Naringin	Cell cycle arrest	G1 cycle arrest by increasing p21 and decreasing survivin in MDA-MB-231 xenograft mice	[25]
	Anticancer metastasis	Suppressed the upregulation of metallopeptidase-9 (MMP-9) and repressed the PI3K/AKT/mTOR/p70S6K signaling pathway	[36]

Hesperetin	Cell cycle arrest	G1-phase cell cycle arrest in human breast cancer MCF-7 cells by downregulating CDK2 and CDK4 together with cyclin D and upregulating p21^Cip1^ and p27^Kip1^	[19]
		Induced the G2/M phase and increased expression of caspase-3, caspase-8, caspase-9, p53, Bax, and Fas death receptor and its adaptor protein Fas-associated death domain-containing protein (FADD) in human cervical cancer SiHa cells	[20]
	Suppress proliferation	Exerted significant inhibitory effect on proliferating cell nuclear antigen in ACF in 1,2-dimethylhydrazine induced colon cancer model in rats	[33]
		Inhibited growth of aromatase-expressing MCF-7 tumor in ovariectomized athymic mice by reducing cyclin D1, CDK4, and Bcl-x(L), while upregulating the level of p57^Kip2^	[34]

Nobiletin	Cell cycle arrest	Blocked cell cycle progression at G1 breast cancer cell lines MDA-MB-435 and MCF-7 and human colon cancer line HT-29	[22]
	Antiangiogenesis	Inhibited angiogenic differentiation induced by VEGF and FGF by downregulation of ERK1/2 and c-JNK and activation of the caspase pathway	[42, 44]

Tangeretin	Cell cycle arrest	Blocked cell cycle progression at G1 breast cancer cell lines MDA-MB-435 and MCF-7 and human colon cancer line HT-29	[22]
	Suppress proliferation	Led to caspase-3 activation and elevated surface phosphatidylserine in human cocon LoVo/Dx cells	[28]
	Anticancer metastasis	Inhibited PGDF-BB-induced proliferation and migration of aortic smooth muscle cells by blocking AKT activation	[38]
	Scavenging of ROS	Inhibited cancer cell proliferation by SOD, CAT, GPx, and nonenzymatic antioxidants and phase II detoxification in 7,12-dimethyl benz(a)anthracene induced mammary carcinoma in rats	[45]

5-Demethyltangeretin (5DT)	Cell cycle arrest	Upregulated p53 and p21^Cip1/Waf1^ and downregulated cdc-2 and cyclin B1 leading to G2/M cell cycle arrest	[23]

Sinensetin	Antiangiogenesis	Inhibited angiogenesis by inducing cell cycle arrest in the G0/G1 phase in HUVEC culture; in zebrafish embryos, it downregulated the mRNA expressions of angiogenesis genes *flt1*, *kdrl*, and *hras *	[43]

5HTMF	Suppress proliferation	Induced cellular apoptosis in human colon cancer cells by p53- and Bax-dependent mechanisms in HCT116 colon cancer cells	[24]
	Cell cycle arrest	Induced cell cycle arrest at G0/G1 phase through a p53- and p21^Cip1/Waf^-dependent mechanism in HCT116 colon cancer cells	[24]

5HPMF	Suppress proliferation	Induced cellular apoptosis in human colon cancer cells by p53- and Bax-dependent mechanisms in HCT116 colon cancer cells	[24]

5HHMF	Cell cycle arrest	Induced G2/M arrest through p53- and p21-independent mechanisms in HCT116 colon cancer cells	[24]
	Suppress proliferation	Induced cellular apoptosis in human colon cancer cells by p53- and Bax-dependent mechanisms in HCT116 colon cancer cells	[24]

Naringenin	Cell cycle arrest	Partly formed an accumulation of cells in the G0/G1 and G2/M phases of the cell cycle in human hepatocellular carcinoma HepG2 cells	[21]
	Anticancer metastasis	Induced heme oxygenase-1(HO-1) expression and subsequently decreased ROS generation and VSMC activation induced by TNF-α	[37]

Hesperidin	Suppress proliferation	Promoted apoptosis via conducting the expression of p53 and PPAR *γ* and suppressing the activation of NF-*κ*B in leukemia cell NALM-6	[27]

Flavonoids extracted from Korean *Citrus aurantium* L.	Cell cycle arrest	Induced non-small-cell lung cancer (NSCLC) A549 cells arrest at the G2/M checkpoint	[18]
	Suppress proliferation	Induced caspase-dependent apoptosis through AKT pathway by inhibiting expression of XIAP and Bcl-2 in human leukemia cells U937	[26]

Gold lotion	Suppress proliferation	In azoxymethane-induced aberrant crypt foci formation, it downregulated the protein levels of iNOS, COX-2, ornithine decarboxylase, VEGF, and matrix metallopeptidase 9 in colonic tissues of mice	[31]
	Anticancer metastasis	Downregulated MMP-2 and MMP-9 protein expression levels and reduced tumor volumes and weights in human prostate tumor xenograft mouse model	[39]
	Antiangiogenesis	Significantly suppressed the protein expression level of VEGF	[39]
		Reduced the protein levels of VEGF in AOM-induced colonic tissues	[31]
